# Special Issue “Cancer Biology: From Genetic Aspects to Treatment: 1st Edition”

**DOI:** 10.3390/ijms27041885

**Published:** 2026-02-15

**Authors:** Silvia Cantara

**Affiliations:** Department of Medical, Surgical and Neurological Sciences, University of Siena, 53100 Siena, Italy; cantara@unisi.it

## 1. Introduction

The first documented description of cancer dates to ancient Egypt in around 1600 BCE and can be found in the Edwin Smith Papyrus [1]—one of the oldest medical texts ever discovered—which reports cases of breast tumors. Several centuries later, in classical Greece, Hippocrates introduced the terms “*καρκίνοι*” for benign lesions and “*καρκινώματα*” for malignant ones [2]. For many centuries, cancer was poorly understood and commonly attributed to imbalances of bodily humors or supernatural causes. An important shift occurred in 1914, when the German biologist Theodor Boveri proposed that cancer could arise from chromosomal abnormalities. In his work “*On the Origin of Malignant Tumors*”, Boveri suggested that defects in mitosis could drive uncontrolled proliferation [3]. For the first time, the origin of cancer was attributed to processes within the cells. This hypothesis was confirmed decades later, with the emergence of molecular biology, which linked cancer development to cellular, genetic, and epigenetic alterations [4,5,6]. This transition marked a fundamental shift from descriptive observation to mechanistic explanation, laying the foundations of modern oncology. Due to advances in our understanding, cancer, which was once considered an incurable disease, has, in many cases, become a treatable condition, with significant improvements in prognosis, therapeutic options, and quality of life [7,8]. Nevertheless, cancer remains challenging. Its marked heterogeneity, biological plasticity, and capacity to adapt to therapeutic drugs continue to limit the efficacy of long-term treatment [9]. Using an integrated approach that involves genome regulation, cellular metabolism, tumor microenvironment, and immune modulation, new and effective strategies for treating cancer can be developed.

With this in mind, we present the first edition of this Special Issue of the *International Journal of Molecular Sciences* entitled “Cancer Biology: From Genetic Aspects to Treatment”, which contains a collection of reviews, original research articles, a hypothesis paper, and a short communication.

We extend our sincere gratitude to the authors, peer reviewers, and editorial team, whose efforts made this Special Issue possible.

To provide a clear overview of the diverse contributions, the articles in this Special Issue can be organized into four interconnected themes: (I) the genetic and epigenetic drivers of malignancy; (II) next-generation diagnostics; (III) the role of signaling and the microenvironment; and (IV) innovative therapeutic strategies aimed at overcoming resistance.

## 2. Genetic and Epigenetic Drivers of Malignancy

Scientists are still investigating the driver mutations at the basis of cancer for both diagnostic and prognostic purposes. In this Special Issue, a short communication reports data on the relationship between innate immune signaling and genetic susceptibility to skin cancer, focusing on single-nucleotide polymorphisms in Toll-like receptor 4 (TLR4). In a case–control cohort, the authors describe a trend of a protective effect of the D299G/T399I TLR4 polymorphism against non-melanoma skin cancer, although statistical significance was not reached. Another study determined the prevalence of pathogenic germline variants in patients with sarcomas, identifying high-to-moderate penetrance gene abnormalities in the *TP53*, *BRCA1*, *SDHA*, *ATM*, and *NBN* genes, again reinforcing the notion that germline testing represents a useful strategy for assessing therapeutic options and personalizing therapies. Along with driver mutations themselves, the timing of their occurrence is also crucial. One experimental study investigates the role of poly(ADP-ribosyl)ation in hepatocyte maturation and its impact on hepatobiliary carcinogenesis. Using a hepatocyte-specific *Parg* mouse knockout model, the authors show that impaired completion of postnatal hepatocyte maturation triggers apoptosis, inflammation, and compensatory regeneration, ultimately creating a premalignant tissue environment. These findings reveal how early developmental and epigenetic perturbations can shape long-term cancer susceptibility.

## 3. Next-Generation Diagnostics and Single-Cell Insights

One study in this Special Issue evaluates the cost-effectiveness of whole-exome sequencing in clinical practice. By systematically comparing sensitivity and specificity across platforms and bioinformatic pipelines, the authors demonstrate that having standardized quality metrics is essential for translating high-throughput sequencing into reliable clinical decisions. Following this line of evidence, another group assessed the performance of an NGS 17-gene panel on thyroid indeterminate fine-needle aspiration cytology, obtaining a sensitivity of 90%, a specificity of 87%, a positive predictive value of 91%, and a negative predictive value of 87% in predicting malignancy. In practice, these data are essential for refining risk stratification and reducing unnecessary surgeries. However, the future of diagnostics lies not only in bulk sequencing but in high-resolution mapping. One in-depth review examines the transformative impact of single-cell sequencing technologies in cancer biology. By resolving intratumoral heterogeneity at genomic and transcriptomic resolution, single-cell approaches have redefined our understanding of clonal evolution, tumor–microenvironment interactions, and therapy resistance. The integration of single-cell data with spatial transcriptomics, artificial intelligence, and multi-omics frameworks is presented as a critical step toward precision oncology, with broad implications for biomarker discovery and patient stratification.

## 4. Signaling and the Tumor Microenvironment

The progression of malignancy is inextricably linked to the molecular dialogue between the tumor and its niche [10,11]. One contribution in this Special Issue provides a comprehensive overview of emerging circulating and tissue biomarkers in esophageal cancer, a malignancy still burdened by late diagnosis and poor prognoses. The review integrates inflammatory mediators, chemokine signaling, extracellular matrix remodeling enzymes, and tight junction proteins with DNA and RNA markers, highlighting how these molecular signatures correlate with tumor stage, metastatic spread, and patient survival. A second review focuses on PARP-1 and PARP-2 as central regulators of metastatic progression, discussing the multifaceted roles of PARP-1 and PARP-2 beyond DNA repair, including the impact of PARP-1 on chemokine signaling, immune modulation, and transcriptional regulation of gene expression, particularly in the contexts of angiogenesis and epithelial-to-mesenchymal transition.

## 5. Innovative Therapeutic Strategies and Overcoming Resistance

Finally, several contributions in this Special Issue focus on translating biological insights into effective treatments. In a study examining the impact of reduced extracellular sodium levels on cisplatin efficacy, the authors demonstrate that hyponatremia promotes chemoresistance across multiple types of cancer cells by activating autophagy and attenuating cytotoxicity. This work provides mechanistic insight into clinical observations linking electrolyte imbalance with poor outcomes and underscores the importance of systemic metabolic conditions in shaping cancer treatment. Another study considered a colorectal cancer model resistant to oxaliplatin. The authors demonstrated that the anthraquinone derivative C2 re-sensitizes resistant cells by modulating the PI3K/AKT/mTOR pathway and simultaneously inducing apoptosis and autophagy. In the search for novel pharmacological agents, the therapeutic potential of diet-derived bioactive compounds is highlighted in a study examining anthocyanins extracted from black soybean coats in hepatocellular carcinoma models. The authors show that these compounds selectively induce apoptosis in cancer cells in a dose-dependent manner through inhibition of the JAK2/STAT3 pathway while leaving normal cells unaffected, supporting the role of natural products as modulators of oncogenic signaling. Hormone-driven signaling pathways are explored in a review dedicated to neurotensin and its receptors in hormone-sensitive cancers. Integrating data from breast, ovarian, and endometrial cancers, the authors show how neurotensin signaling intersects with estrogen regulation to promote proliferation, invasion, and disease progression. The review also highlights the prognostic value of neurotensin receptor expression and discusses emerging therapeutic opportunities, including radioligand and theranostic approaches, positioning this pathway as a promising target within personalized oncology. An intriguing hypothesis published in this Special Issue proposes that aberrant transcription of pericentromeric alpha satellite repeats contributes to heterochromatin dysfunction, genome instability, and prostate cancer progression. The authors suggest that the stable controlled transcription of satellite DNA might be important in terms of controlling disease development and that prostate cancer can be treated by interfering with epigenetic aspects in combination with other more conventional therapies. Finally, metabolic plasticity is analyzed in a study profiling three distinct *TP53*-mutant esophageal adenocarcinoma cells (OE33, OE19, and FLO1) representing progressive stages of tumor differentiation and harboring distinct *TP53* alterations. The analyses revealed different metabolic phenotypes associated with *TP53* status that, according to the authors, offer novel therapeutic avenues for personalized treatment in esophageal cancer.

In conclusion, the journey from the first ancient descriptions to today’s knowledge of cancer genetics highlights our incredible progress and reminds us of the work still ahead. We hope this Special Issue provides not only valuable data but also the inspiration to keep pushing the boundaries of what is possible in cancer care to move us one step closer to a future of personalized therapy.
